# Impact of Transient and Persistent Donor-Specific Antibodies in Lung Transplantation

**DOI:** 10.3389/ti.2024.12774

**Published:** 2024-05-08

**Authors:** S. Auner, C. Hillebrand, P. M. Boehm, J. Boecker, D. Koren, S. Schwarz, Z. Kovacs, G. Murakoezy, G. Fischer, C. Aigner, K. Hoetzenecker, P. Jaksch, A. Benazzo

**Affiliations:** ^1^ Vienna Lung Transplant Program, Department of Thoracic Surgery, Medical University of Vienna, Vienna, Austria; ^2^ Department of Blood Group Serology and Transfusion Medicine, Medical University of Vienna, Vienna, Austria

**Keywords:** donor specific antibody (DSA), humoral rejection, lung transplantation, AMR, antibody-mediated rejection

## Abstract

Lung transplantation (LuTx) is an established treatment for patients with end-stage lung diseases, however, outcomes are limited by acute and chronic rejection. One aspect that has received increasing attention is the role of the host’s humoral alloresponse, particularly the formation of *de novo* donor-specific antibodies (dnDSAs). The aim of this study was to investigate the clinical significance of transient and persistent dnDSAs and to understand their impact on outcomes after LuTx. A retrospective analysis was conducted using DSA screening data from LuTx recipients obtained at the Medical University of Vienna between February 2016 and March 2021. Of the 405 LuTx recipients analyzed, 205 patients developed dnDSA during the follow-up period. Among these, 167 (81%) had transient dnDSA and 38 (19%) persistent dnDSA. Persistent but not transient dnDSAs were associated with chronic lung allograft dysfunction (CLAD) and antibody-mediated rejection (AMR) (*p* < 0.001 and *p* = 0.006, respectively). CLAD-free survival rates for persistent dnDSAs at 1-, 3-, and 5-year post-transplantation were significantly lower than for transient dnDSAs (89%, 59%, 56% vs. 91%, 79%, 77%; *p* = 0.004). Temporal dynamics of dnDSAs after LuTx have a substantial effect on patient outcomes. This study underlines that the persistence of dnDSAs poses a significant risk to graft and patient survival.

## Graphical Abstract

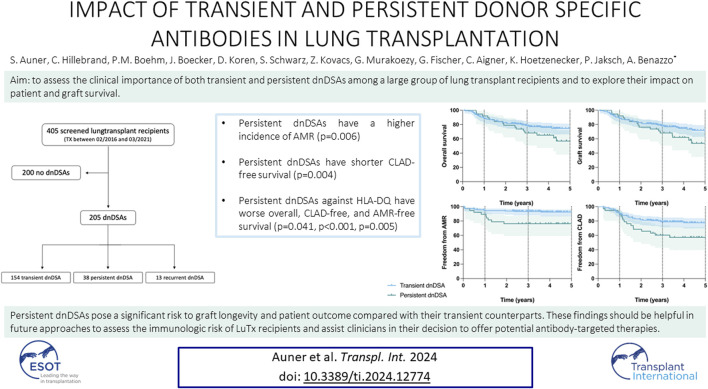

## Introduction

Lung transplantation represents a life-saving therapeutic option for patients with end-stage lung diseases, however, outcomes remain impaired by acute and chronic rejection. Over the last decade, there has been growing recognition of the pathogenic significance of the host’s humoral response against the pulmonary allograft in addition to cellular immunity. It has been observed that patients with antibody-mediated rejection who survive the acute phase often develop long-term structural derangements of the allograft, leading to CLAD [1, 2]. However, to date, dnDSAs without clinical signs of graft dysfunction are not considered a stringent indication for treatment, primarily because currently available therapeutic interventions carry significant associated risks.

Few studies have already shown the pathogenic role of persistent DSAs. Schmitzer et al. drew attention to the contrasting outcomes linked with transient versus persistent DSAs, and showed a significantly reduced survival in patients with persistent DSAs [3]. Lobo et al. highlighted the significant correlation between DSA presence, particularly with anti-HLA DQ specificity, and increased AMR and CLAD [4].

We hypothesized that transient dnDSAs, which may appear briefly after transplantation, lack clinical significance. Persistent dnDSAs, on the other hand, reflect an ongoing subclinical humoral response against the graft. The primary aim of this study was to assess the clinical importance of both transient and persistent dnDSAs among a large group of lung transplant recipients and to explore their impact on patient and graft survival.

## Materials and Methods

### Study Design

**FIGURE 1:**
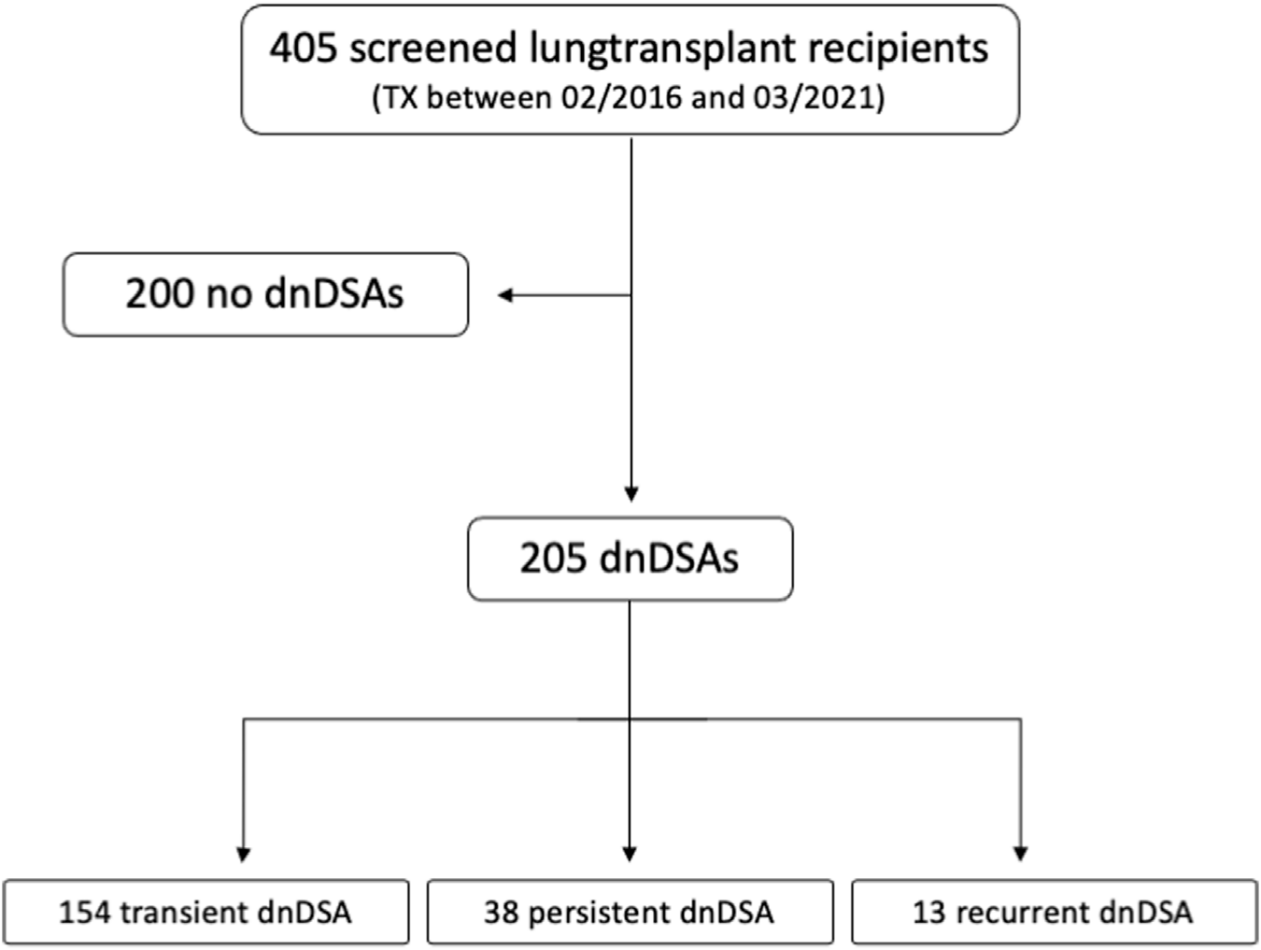
Flowchart showing patient selection.

This study was a retrospective single-center analysis of data obtained at the Medical University of Vienna between February 2016 and March 2021. We reviewed DSA screening data from 405 lung transplant recipients. The analysis included adult patients with *de novo* DSAs after primary transplantation. Patients who underwent retransplantation or multi-organ transplantation were excluded from the study (as shown in Figure 1).

This study has been approved by the Institutional Ethical Committee of the Medical University of Vienna [EK-Nr. 1899/2023] and was performed according to the Declaration of Helsinki. Due to the retrospective nature of the study patient consent was waived.

### Pretransplant Screening

Prior to transplantation, all patients underwent screening for potential pre-sensitization using the complement-dependent lymphoma cytotoxicity assay and the Luminex multiplex assay. If positive, a Single Antigen bead assay (LABScreen® Single Antigen; OneLambda) was performed. Based on the results of the Single Antigen bead assay, unacceptable antigens (UAGs) were defined based on MFI > 10,000 and on clinical plausibility, i.e., previous sensitization events (e.g., pregnancies). When UAGs were present, the virtual panel reactive antibody score (vPRA) was calculated using the “Eurotransplant Reference Laboratory virtual PRA Calculator.”^1^ On the day of transplantation, a single antigen bead assay was performed. Based on this assay, antibodies present with an MFI > 1,000 were classified as “preformed” DSAs. Antibodies post-transplant with an MFI > 1,000, which were not detected or were below this threshold before transplant, were classified as *de novo* DSAs.

Flow cytometric crossmatch (FCXM) was conducted for every patient immediately after transplantation. Donor lymphocytes were incubated with the patient’s serum, along with both negative and positive controls. Differently from other centers that might employ the median channel shift of median MFI for analysis, our center utilizes a linear acquisition approach. Accordingly, an FCXM test is deemed positive when the fluorescence intensity measurement exceeds 6,000 units above the mean of the negative controls.

### Clinical Protocol

Upon arrival at the intensive care unit, patients either received either a single 30 mg dose of alemtuzumab (Genzyme/Sanofi, Cambridge, United States), anti-thymocyte globulin (ATG, Neovii, Rapperswil-Jona, Switzerland) or no induction therapy after transplantation. If alemtuzumab therapy was given, a low-dose maintenance immunosuppression protocol based on tacrolimus and steroids was followed for the first year, with the addition of mycophenolate mofetil from the second year onward [5]. Otherwise, patients received a standard triple-drug maintenance immunosuppression. Since 2009 our center routinely used alemtuzumab as induction therapy agent. Since 2009, however, the induction policy changed over time. At the beginning of our experience, alemtuzumab was not administered in patients with multi-resistant bacteria, in sensitized patients or in patients with connective-tissue diseases (CTD). Patients with multi-resistant bacteria did not receive any induction, while sensitized or CTD patients received ATG. However, due to the excellent results with alemtuzumab, sensitized and CTD patients have been treated with alemtuzumab for approximately 5 years. More details regarding our induction and immunosuppression strategies have been published elsewhere [6]*.* All LuTx recipients received lifelong pneumocystis prophylaxis with trimethoprim-sulfamethoxazole or atovaquone. Inhalation therapy with Amphotericin B was administered for a minimum of 3 months. For cytomegalovirus (CMV) prevention, patients received CMV hyperimmunoglobulins and valganciclovir for at least 3 months, while patients identified as high-risk (donor CMV positive, recipient CMV negative) receiving this prophylaxis for an extended period of up to 1 year.

### Follow-Up

Surveillance bronchoscopy with transbronchial biopsy (TBB) and bronchoalveolar lavage (BAL) were scheduled at 2 weeks, 1-, 2-, 3-, 6-, and 12-month post-transplant. TBBAs were graded according to the latest ISHLT criteria [7]. Annual lung CT scans were part of the follow-up protocol. The diagnosis of CLAD was attributed by two independent transplant pulmonologists following the ISHLT consensus report guidelines [8]. If a patient’s lung function deteriorated without reversible reasons, patients received 250 mg azithromycin three times weekly for at least 3 months. If the lung function continued to decline, extracorporeal photopheresis (ECP) was initiated.

According to the ISHLT, clinical antibody-mediated rejection (AMR) was identified by the presence of donor-specific antibodies, pathological signs of tissue injury, complement activation (evidenced by c4d deposition), or detectable graft dysfunction [2]. Acute cellular rejection (ACR) was histologically graded based on the intensity of cellular infiltrates and fibrosis found in transbronchial biopsies and ranged from grade A0 (no acute rejection) to A4 (severe acute rejection) [9].

### Measurement of Post-Transplant Donor Specific Antibodies

HLA class I and class II specific antibodies in patients’ sera were detected using single antigen beads (SAB) (LABScreen Single Antigen HLA Class I and Class II, One Lambda, Canoga Park, CA, United States) on the Luminex Flexmap 3D platform (Luminex Corporation, Austin, TX, United States). The assay was performed according to the manufacturer’s protocol with minor modifications. Sera were subjected to pretreatment with ethylenediaminetetraacetic acid (EDTA) at a final concentration of 50 mM to avoid a prozone effect, followed by incubation with the beads. After three wash phases, a fluorescence-labelled IgG antibody was added and further incubated. After three additional wash steps, fluorescence intensity of the beads was measured. The observed mean fluorescence intensity (MFI) of the beads was reported after subtracting the value of the negative control bead (normalized values). An MFI value > 1,000 was considered positive. Initially, the sera were evaluated in their undiluted form. However, if MFI values of the beads exceeded 20,000, implying antibody saturation of the beads, the serum was diluted with PBS prior to reassessment. In this case, the MFI values were reported as the initial observed values multiplied by the dilution factor. Measurements were analyzed using the HLA Fusion software (Thermo Fisher Scientific Inc.). DSAs were screened at every follow-up visit at 2 weeks, 1-, 2-, 3-, 6-, and 12-month post-transplantation and in case of clinical deterioration. DnDSAs were classified as “transient” when they were detected for a period of less than 6 months following transplantation. Conversely, dnDSAs were classified as “persistent” when their presence extended for 6 months or longer. Recurrent DSAs were defined as circulating dnDSAs, which disappeared without treatment and later reemerged*.* Based on their initial detection post-transplantation, dnDSAs were further categorized into “early” dnDSAs (emerging within the first 6 months) and “late” dnDSAs (manifesting after 6 months). Subsequently, dnDSAs were classified into subgroups according to their MFI value: MFI class I: 1,000–2,000, MFI class II: 2,000–5,000, MFI class III: 5,000–10,000, MFI class IV: >10,000. For further comparative analysis, a “*mean MFI intensity score*” was computed for each individual, calculated as the average MFI class of all dnDSAs identified in that particular patient.

### Statistical Analysis

Categorical variables are expressed as absolute and relative frequencies and were compared using a chi-square test. Continuous variables were expressed as median and interquartile range (IQR) or mean and standard deviations. Chi-square tests, Fisher exact tests, Mann-Whitney U-tests, or ANOVA were used to compare variables as applicable. Patient and graft survival as well as freedom from AMR and CLAD were displayed with Kaplan-Meier-curves and compared using a log-rank test. Univariate and multivariable Cox regression were performed to find risk factors for mortality and CLAD. Variables that reached significance in the univariate analyses, they were included in a multivariable Cox regression. Data was analyzed using SPSS version 27.0 software, graphics were designed with GraphPad Prism 6.

## Results

Within the study period, we analyzed data from 405 LuTx recipients. Of these patients, 205 developed dnDSAs during the median follow-up period of 3.44 years (IQR 2.45–4.81) and were included in the present study. 167 (81%) patients accounted for the transient dnDSA group and 38 (19%) for the persistent dnDSA group. The median age at the time of transplantation was 56 years (IQR: 42–62) and 36% of the cohort were female. 202 (99%) patients underwent a double lung transplantation (DLuTx), while only three received a single lung transplantation (SLuTx). Chronic obstructive pulmonary disease (COPD) was the most common underlying diagnosis, accounting for 41% of the cohort. Detailed patient characteristics, including patients with recurrent dnDSAs, are summarized in Table 1. Patient characteristics for transient, persistent, and recurrent dnDSAs are summarized in Supplementary Table S2.

**TABLE 1:** Patient characteristics for patients with transient and persistent dnDSA.

Patients’ characteristics					
		Overall (*n* = 205)	Transient (*n* = 167)	Persistent (*n* = 38)	*p*-value
Female (*n*, %)		73 (36%)	67 (40%)	6 (16%)	0.005
Age in years (median, IQR)		56 (42–62)	57 (44–62)	53 (35–60)	0.11
Type of TX (*n*, %)	DLuTx	202 (99%)	165 (99%)	37 (97%)	0.5
	SLuTx right	2 (1.0%)	1 (0.6%)	1 (2.6%)	
	SLuTx left	1 (0.5%)	1 (0.6%)	0 (0%)	
Underlying diagnosis (n, %)	Obstructive	84 (41%)	72 (44%)	12 (31%)	0.13
	Restrictive	65 (32%)	52 (31%)	13 (34%)	
	Vascular	9 (4.4%)	9 (5.4%)	0 (0%)	
	Suppurative	38 (19%)	27 (16%)	11 (29%)	
	Others	9 (4.4%)	7 (4.2%)	2 (5.3%)	
UAGs (*n*, %)		11 (5.9%)	10 (6.4%)	1 (3.1%)	0.7
Crossmatch positive (*n*, %)		1 (0.5%)	1 (0.6%)	0 (0%)	>0.9
High grade ACR (*n*, %)		8 (3.9%)	4 (2.4%)	4 (11%)	0.041
High grade LB (*n*, %)		9 (4.4%)	5 (3.0%)	4 (11%)	0.063
Immuno-suppression (*n*, %)	Ciclosporin	4 (2.0%)	4 (2.4%)	0 (0%)	>0.9
	Tacrolimus	198 (98%)	161 (98%)	37 (100%)	
CMV risk (*n*, %)	D+/R−	58 (28%)	42 (25%)	16 (42%)	0.079
	D+/R+	68 (33%)	61 (37%)	7 (18%)	
	D−/R+	58 (28%)	45 (27%)	13 (34%)	
	D−/R−	20 (9.8%)	18 (11%)	2 (5.3%)	
AMR (*n*, %)		23 (11%)	14 (8.4%)	9 (24%)	0.018
HLA class I (*n*, %)		133 (65%)	106 (63%)	27 (71%)	0.4
HLA class II (*n*, %)		143 (70%)	108 (65%)	35 (92%)	<0.001
DSA against HLA—A (*n*, %)		69 (34%)	51 (31%)	18 (47%)	0.048
DSA against HLA—B (*n*, %)		75 (37%)	55 (33%)	20 (53%)	0.023
DSA against HLA—C (*n*, %)		43 (21%)	33 (20%)	10 (26%)	0.4
DSA against HLA—DQ (*n*, %)		116 (57%)	84 (50%)	32 (84%)	<0.001
DSA against HLA—DP (*n*, %)		18 (8.8%)	13 (7.8%)	5 (13%)	0.3
DSA against HLA—DR (*n*, %)		56 (27%)	40 (24%)	16 (42%)	0.023
mean MFI intensity score (median, IQR)		2.00 (1.25–2.40)	2.00 (1.00–2.00)	2.53 (2.00–2.96)	<0.001
CLAD (*n*, %)		52 (25%)	35 (21%)	17 (45%)	0.002

Abbreviations: TX, transplantation; DLuTx, double lung transplantation; SLuTx, single lung transplantation; HLA, human leukocyte antigen; UAG, unacceptable antigen; ACR, acute cellular rejection; LB, lymphocytic bronchiolitis; D, donor; R, recipient; AMR, antibody mediated rejection; DSA, donor specific antibody; MFI, mean fluorescence intensity; CLAD, chronic lung allograft dysfunction.

### Pretransplant Immunization

Eleven patients (5.9%) had UAGs with a mean vPRA of 42% (range 1%–98%). Characteristics of those 11 patients are displayed in Table 2. All presensitized patients received an organ matched on all 6 HLA donor-recipient loci.

**TABLE 2:** Patients with unacceptable antigens.

Patients with UAGs										
Patients	UAGs	vPRA (%)	Matched organ	preTX therapy	postTX therapy	Crossmatch	Induction	dnDSAs	AMR	CLAD
patnr001	B17	8	Yes	No	No	Negative	None	Transient	No	No
patnr002	B12, Dr13, DQ1	78	Yes	ECP	No	Negative	Alemtuzumab	Transient	No	No
patnr003	A2	51	Yes	ECP	No	Negative	Alemtuzumab	Transient	No	No
patnr004	A2, B17, DR4, DR53	72	Yes	ECP+IAS	No	Negative	ATG	Transient	No	No
patnr005	B46, B73, Cw7, Cw8, Cw3, C*12, C*16	81	Yes	ECP	No	Negative	ATG	Transient	Yes	No
patnr006	B18, DR4, DR6, DR2, DR3, DR52, DQ1	98	Yes	ECP	No	Negative	ATG	Transient	No	No
patnr007	B12	23	Yes	No	No	Negative	Alemtuzumab	Transient	No	Yes
patnr008	A25, B22, Cw3	5	Yes	No	No	Negative	Alemtuzumab	Persistent	No	No
patnr009	B12	23	Yes	No	No	Negative	Alemtuzumab	Transient	No	Yes
patnr010	DR7	23	Yes	No	No	Negative	Alemtuzumab	Transient	No	No
patnr011	A80	1	Yes	No	No	Negative	ATG	Transient	No	No

Abbreviations: vPRA, virtual panel reactive antibodies; preTX, pretransplantation; postTX, post-transplantation; ECP, extracorporeal photopheresis; IAS, immunoadsorption; ATG, anti-thymocyte globulin.

Only one patient in the cohort had a positive FCXM. This patient, diagnosed with usual interstitial pneumonia (UIP) and rheumatoid arthritis had previously been treated with rituximab. The patient was bridged to transplantation on veno-venous ECMO. Prior to transplantation, the patient was negative for both HLA class I and II in the single antigen bead assay. The patient developed dnDSAs and clinical AMR 190 days after transplantation, which was treated with ECP, IAS and ATG. The patient died 26 days after AMR diagnosis due to respiratory failure.

### DSA Profile

A complete list and median MFI class of all timepoints for each dnDSA based on the Luminex Single Antigen bead assay is shown in Supplementary Table S1. After transplantation, 65% of the overall cohort (*n* = 133) tested positive for HLA class I antibodies with a slightly higher proportion in the persistent group (71%) than in the transient group (63%). This difference was not statistically significant (*p* = 0.4). A more notable discrepancy was observed for DSA class II, for which 70% of the total cohort (*n* = 143) tested positive. Of these patients, 92% accounted for the persistent group and 65% for the transient group (*p* < 0.001).

When assessing the frequency of specific transient or persistent dnDSAs, significant variations were evident for dnDSAs against HLA-A, -B, -DQ and -DR (*p* = 0.048, *p* = 0.023, *p* < 0.001, and *p* = 0.023, respectively) (Table 1).

The median of the *mean MFI intensity score* was significantly higher in the persistent DSA group with a score of 2.53 (IQR: 2.00–2.96), than in the transient group with a score of 2.00 (IQR: 1.00–2.00) (*p* < 0.001).

Next, we analyzed the impact transient and persistent dnDSAs against a specific HLA subclass on AMR and CLAD development. Patients with persistent dnDSA against HLA-DQ had a higher incidence of AMR (*p* = 0.004) and CLAD (*p* = 0.002). Furthermore, persistent dnDSA against HLA-A showed a significantly higher rates of AMR, but not CLAD (*p* = 0.046). Patients with persistent dnDSA against HLA-DQ showed a significantly worse overall, CLAD-free, and AMR-free survival (*p* = 0.041, *p* < 0.001, and *p* = 0.005, respectively), whereas persistent dnDSAs against HLA-A had a significantly worse overall and AMR free survival (*p* = 0.020 and *p* = 0.012). Specifically, persistent dnDSA against HLA-DQB had a significantly worse overall, CLAD-free, and AMR-free survival (*p* = 0.026, *p* = 0.007, and *p* = 0.016, respectively).

### Rejections and Long-Term Survival

**FIGURE 2:**
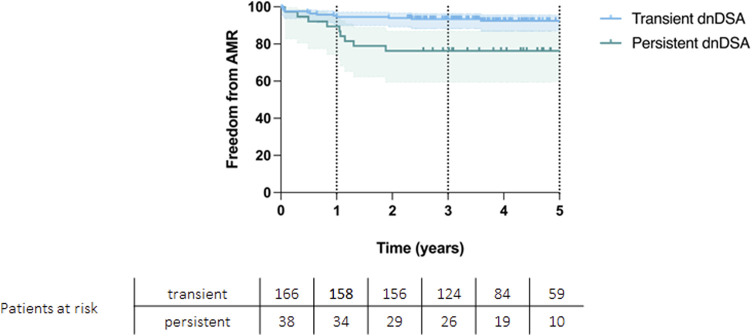
Kaplan-Meier curve showing AMR-free survival for patients with transient and persistent DSAs (*p* = 0.004).

Eleven percent (*n* = 22) of the entire cohort developed an AMR. Within the persistent dnDSA group, the incidence was 24% (*n* = 9) as compared to 7.8% (*n* = 13) in the transient group (*p* = 0.008). Patients with AMR had significantly worse survival than those without AMR episodes (*p* < 0.001). AMR free survival rates at 1, 3 and 5 years of 89%, 76%, and 76% for persistent DSA group and 95%, 93%, and 92% for transient DSA group (*p* = 0.004, Figure 2). Patients with AMR, that received any form of treatment are displayed in Supplementary Table S3.

ACR free survival rates at 1, 3 and 5 years were 99%, 97% and 97% for the transient group and 95%, 89% and 89% for the persistent group (*p* = 0.021).

**FIGURE 3:**
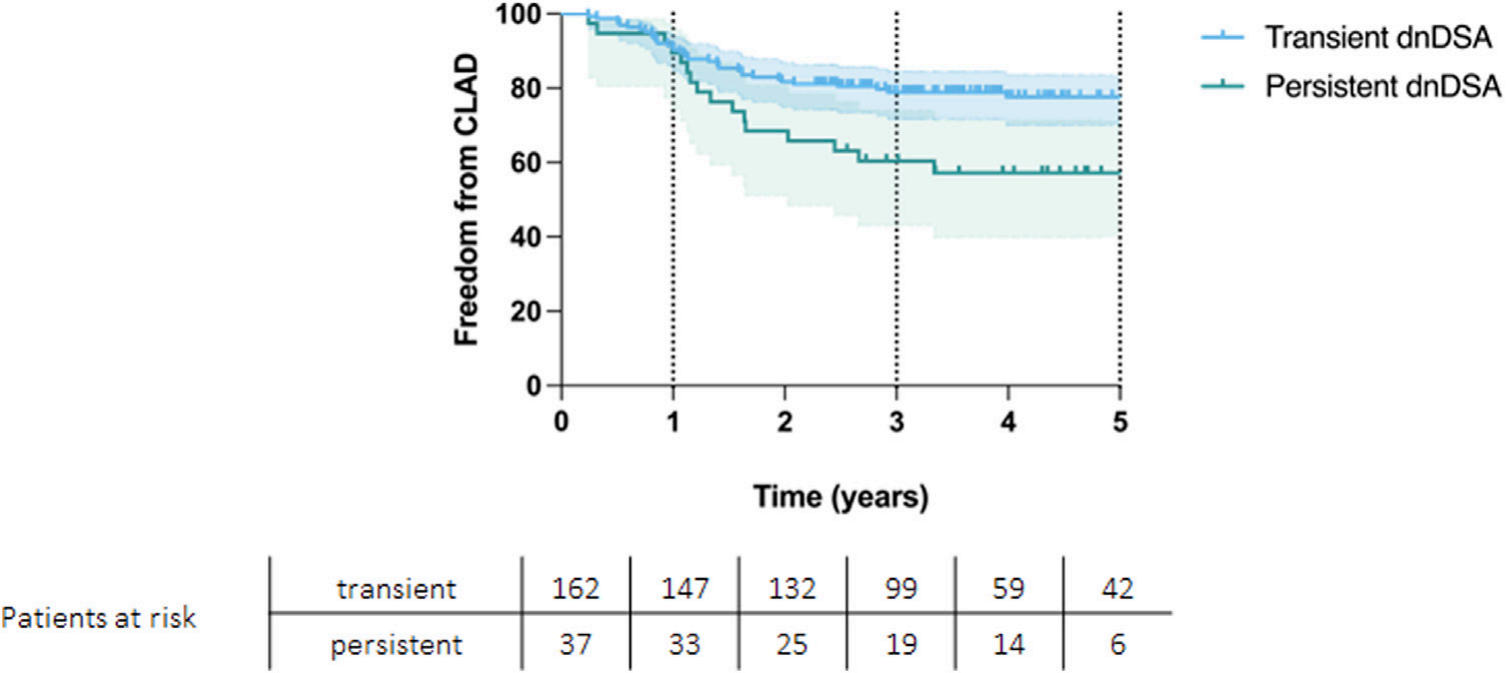
Kaplan-Meier curve showing CLAD-free survival for patients with transient and persistent DSAs (*p* = 0.004).

CLAD-free survival significantly differed between the groups, with CLAD free survival rates at 1, 3 and 5 years of 89%, 59%, and 56% for persistent DSA group and 91%, 79%, and 77% for transient DSA group (*p* = 0.004, Figure 3).

**FIGURE 4:**
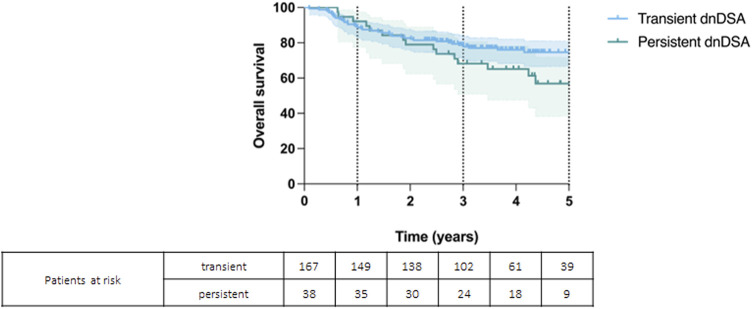
Kaplan-Meier curve showing overall survival for patients with persistent and transient *de novo* DSAs (*p* = 0.074).

**FIGURE 5:**
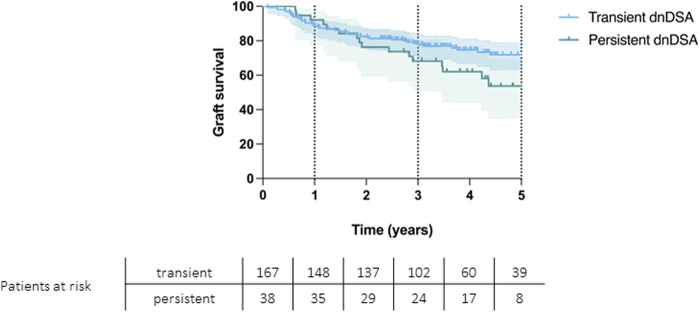
Kaplan-Meier curve showing graft survival for patients with persistent and transient *de novo* DSAs (*p* = 0.069).

Patients’ overall survival rates at 1, 3 and 5 years were 92%, 68%, and 57% for persistent DSAs and 89%, 79%, and 75% for transient DSAs (*p* = 0.074) (Figure 4). Graft survival rates at 1, 3, and 5 years were 92% 68% and 54% for persistent DSAs and 89%, 78%, and 71% for transient DSAs (*p* = 0.069) (Figure 5).

### Risk Factor Analysis

Univariate and multivariable cox regression for overall survival and CLAD occurrence were performed to identify risk factors. AMR was identified as the only independent risk factors for impaired survival (*p* < 0.001) and for CLAD occurrence (*p* = 0.030) in the multivariable cox regression model (Table 3). Interestingly, dnDSAs against HLA-DQ, were not identified as risk factors in both adjusted models. To elucidate the potential impact of MFI intensity on outcomes, we analyzed MFI classes one to four in our risk factor analysis. While MFI class 1 exhibited a protective trend against mortality in the univariate analysis, it did not reach statistical significance in the multivariate model. Similarly, MFI class 4 appeared to be a risk factor for CLAD occurrence in the univariate analysis, yet this did not remain significant in the multivariate analysis.

**TABLE 3:** Risk factor analysis for mortality and CLAD occurrence.

Univariate cox regression for mortality					Multivariable cox regression for mortality			
Variable	HR	CI		*p*-value	HR	CI		*p*-value
		Lower limit	Upper limit			Lower limit	Upper limit	
Age	**1.02**	**1.002**	**1.04**	**0.040**	**1.03**	**1.01**	**1.05**	**0.008**
Female	1.01	0.98	1.04	0.421				
Preformed DSA	0.83	0.37	1.82	0.636				
DSAclass I	1.16	0.67	2.02	0.586				
DSAclass II	0.98	0.55	1.73	0.946				
persistent dnDSA	1.69	0.94	3.01	0.078				
MFI class 4	1.69	0.94	3.05	0.082				
MFI class 3	0.98	0.51	1.89	0.943				
MFI class 2	1.39	0.82	2.34	0.223				
MFI class 1	**0.32**	**0.14**	**0.75**	**0.008**	0.38	0.14	1.03	0.061
dnDSA against HLA—A	1.05	0.61	1.80	0.871				
dnDSA against HLA—B	1.48	0.87	2.51	0.151				
dnDSA against HLA—C	1.15	0.62	2.14	0.653				
dnDSA against HLA—DP	0.96	0.38	2.40	0.925				
dnDSA against HLA—DQ	1.10	0.65	1.86	0.793				
dnDSA against HLA—DQA	1.14	0.62	2.09	0.668				
dnDSA against HLA—DQB	1.00	0.60	1.69	0.996				
dnDSA against HLA—DR	1.48	0.85	2.57	0.167				
Persistent dnDSA against HLA—A	7.99	1.00	34.88	0.059				
Persistent dnDSA against HLA—B	0.05	0.01	18.27	0.551				
Persistent dnDSA against HLA—C	1.21	0.16	9.34	0.853				
Persistent dnDSA against HLA—DQ	**2.00**	**1.01**	**3.93**	**0.046**	0.834	0.17	4.05	0.822
Persistent dnDSA against HLA—DQA	1.73	0.54	5.53	0.359				
Persistent dnDSA against HLA—DQB	**2.34**	**1.08**	**5.04**	**0.031**	2.03	0.36	11.56	0.424
Persistent dnDSA against HLA—DR	1.96	0.77	4.97	0.156				
AMR	**4.33**	**2.42**	**7.74**	**<0.001**	**4.14**	**2.11**	**8.12**	**<0.001**
Mean MFI intensity score	**1.64**	**1.17**	**2.22**	**0.003**	0.93	0.58	1.48	0.742

Univariate cox regression for CLAD occurrence					Multivariable cox regression for CLAD occurrence			
Variable	HR	CI		*p*-value	HR	CI		*p*-value
		Lower limit	Upper limit			Lower limit	Upper limit	
Age	0.99	0.98	1.02	0.831				
Female	0.81	0.45	1.46	0.487				
DSAclass I	1.06	0.60	1.88	0.586				
DSAclass II	0.89	0.50	1.61	0.741				
Persistent dnDSA	**2.30**	**1.29**	**4.11**	**0.005**	0.60	0.08	4.64	0.621
MFI class 4	**2.57**	**1.44**	**4.59**	**0.001**	1.43	0.65	3.14	0.372
MFI class 3	0.80	0.39	1.65	0.549				
MFI class 2	0.78	0.44	1.39	0.395				
MFI class 1	0.63	0.31	1.29	0.206				
dnDSA against HLA—A	0.84	0.46	1.51	0.561				
dnDSA against HLA—B	1.29	0.69	2.41	0.429				
dnDSA against HLA—C	1.13	0.61	2.19	0.690				
dnDSA against HLA—DP	1.06	0.61	1.85	0.837				
dnDSA against HLA—DQ	0.57	0.18	1.82	0.325				
dnDSA against HLA—DQA	0.89	0.46	1.74	0.737				
dnDSA against HLA—DQB	1.06	0.62	1.83	0.829				
dnDSA against HLA—DR	1.34	0.75	2.39	0.327				
Persistent dnDSA against HLA—A	0.05	0.01	20.23	0.738				
Persistent dnDSA against HLA—B	0.05	0.01	34.62	0.593				
Persistent dnDSA against HLA—C	0.97	0.13	7.43	0.973				
Persistent dnDSA against HLA—DQ	**3.25**	**1.61**	**6.58**	**0.001**	6.23	0.53	73.64	0.147
Persistent dnDSA against HLA—DQA	3.07	0.89	10.53	0.075				
Persistent dnDSA against HLA—DQB	**2.82**	**1.29**	**6.19**	**0.010**	0.58	0.13	2.55	0.470
Persistent dnDSA against HLA—DR	2.48	0.81	7.63	0.113				
AMR	**3.10**	**1.63**	**5.92**	**<0.001**	**2.20**	**1.08**	**4.48**	**0.030**
Mean MFI class score	**1.64**	**1.15**	**2.35**	**0.006**	1.24	0.78	1.97	0.368

Abbreviations: CLAD, chronic lung allograft dysfunction; DSA, donor specific antibodies; dnDSA, *de-novo* donor specific antibodies; HLA, human leukocyte antigen; AMR, antibody mediated rejection; MFI, mean fluorescence intensity.

Bold values are statistically significant.

## Discussion

The current study provides valuable insights into the temporal dynamics of *de novo* donor-specific antibodies after lung transplantation and their potential role in allograft dysfunction. The primary aim of this study was to assess the clinical importance of both transient and persistent dnDSAs and to explore their impact on patient and graft survival. We could demonstrate that CLAD-free and AMR-free survival was significantly higher in patients with transient DSAs, signaling the potential negative impact of persistent dnDSAs on outcomes after LuTx.

DSAs have been associated with glomerulopathy in renal transplant recipients and cardiac allograft vasculopathy in cardiac transplant recipients [10, 11]. Similarly, observational studies in LuTx suggested that dnDSAs could have a deleterious effect on survival and CLAD [12–16]. In addition, some publications report a beneficial effect of treating dnDSAs in the absence of graft dysfunction. For example, a single-center retrospective study analyzed the effects of preemptive treatment of early DSAs with IVIG and showed comparable graft survival in patients receiving preemptive treatment compared to patients without DSAs [17]. Hachem et al performed a prospective cohort study showing that patients who developed DSAs and received antibody-directed therapy had similar rates of CLAD and acute rejection as patients without DSAs [18]. Finally, in a recent multicenter retrospective analysis, Keller et al provided evidence that asymptomatic patients with dnDSAs who received preemptive treatment of any kind had a lower risk of CLAD or death than untreated patients with dnDSAs [19]. Based on these findings, it is meaningful to speculate that an active approach towards patients with dnDSAs could result in improved outcomes. Nevertheless, there is only limited evidence on the efficacy of available therapies and most of them are associated with a high-risk side effect profile. As a consequence, treating asymptomatic patients with dnDSAs remains clinically and ethically questionable and it is of paramount importance to identify which patients could profit from such an aggressive approach.

In particular, patients with persistent antibodies have worse freedom from AMR and CLAD compared to patients with transient dnDSAs. Our findings confirm previous observations. Schmitzer et al. investigated the relevance of DSA prospectively in 72 patients and showed that persistent DSAs had a significantly reduced survival compared to transient or no DSAs [3]. Especially patients developing AMR had dramatic outcomes in our cohort. These patients could benefit from antibody-directed therapies and further prospective studies should aim to assess possible strategies in this high-risk cohort. Furthermore, our analysis of MFI intensity classes suggests its role is not straightforward and may be moderated by other factors. The potential collinearity between higher MFI classes and persistent dnDSA groups was considered, however, our analysis indicates that the relationship between MFI intensity, dnDSA persistence, and clinical outcomes is complex and warrants further investigation.

Since 2016, our center has started to routinely screen patients for dnDSAs, before 2016, patients were only tested in case of functional decline or clinical suspicion of rejection. This practice is not common in every transplant program yet, partly explaining the high variability in the reported incidence of dnDSAs [15, 20, 21]. Starting a screening program had significantly affected our current practice. Indeed, dnDSA were present in more than half of the study population and one-third of them were persistent. Moreover, based on the current findings, it is meaningful to argue that a large proportion of these patients would require some antibody-directed therapies, which can on one hand improve long-term outcomes and on the other hand, reduce healthcare costs by decreasing future hospitalization, intensive supportive care or more expensive treatments.

In the follow-up period, the majority of dnDSA were directed against antibodies of HLA class II antigens. Especially antibodies against HLA-DQ antigens were significantly higher in the persistent group than in the transient group. Remarkably, the development of persistent dn-DSA-DQ was significantly associated with a higher incidence of CLAD and AMR. This association has also been found in other retrospective single-center studies [4, 22, 23]. For example, Tikkanen et al. showed that recipients with *de novo* DSAs against HLA-DQ had an increased risk for developing CLAD [22]. Also, Roux et al. analyzed data from 206 LuTx recipients with and without AMR. They showed that the DSA-DQ was associated with AMR and CLAD [23].

We acknowledge that our study is not free of limitations. Given the retrospective design of our study and the limited number of observations, there is a potential for miscoded data and an increased risk of Type I error. Then, different induction therapies have been used in the study cohort, which might complicate the interpretation of the results. Furthermore, different desensitization strategies as well as multiple AMR treatments have been implied in our center overtime. This reflects the lack of efficient treatment but can represent possible confounders. We furthermore must acknowledge the lack of sequential screening pretransplant after potentially sensitizing exposures as a limitation. Another limitation of our study is the lack of additional confirmatory testing for atypical DQ bead reactions, despite the known potential for artifacts in bead array assays. Finally, the definition used to distinguish transient from persistent dnDSAs is based on our clinical experience and past literature, instead of being based on robust mechanistic data.

In summary, our study demonstrated that persistent dnDSAs pose a significant risk to graft longevity and patient outcome compared with their transient counterparts. These findings should be helpful in future approaches to assess the immunologic risk of LuTx recipients and assist clinicians in their decision to offer potential antibody-targeted therapies. Finally, our results may provide a rationale starting point on defining a high-risk population that ought to be included in future randomized controlled intervention trials.

## Data Availability

The data analyzed in this study is subject to the following licenses/restrictions: The dataset are property of the Medical University of Vienna. Access to the dataset can be provided after formal approval of the legal departments of the three involved centers and the of the first and last authors. Requests to access these datasets should be directed to rechtsabteilung@meduniwien.ac.at.

## Footnotes

1
https://www.etrl.org/InformationVPRA.aspx
