# Supplementary material for: Impact of Transient and Persistent Donor-Specific Antibodies in Lung Transplantation
Source: Transpl Int. 2024 May 8;37:12774. doi: 10.3389/ti.2024.12774 (PMC11110840; doi:10.3389/ti.2024.12774)
Supplement: Supplementary file 1 [file Table1.docx]

Supplementary table 1: List of all dnDSA

| dnDSAs | persistent DSAs | | | transient DSAs | | | recurrent DSAs | | |
| --- | --- | --- | --- | --- | --- | --- | --- | --- | --- |
|  | n | median MFI class | IQR | n | median MFI class | IQR | n | median MFI class | IQR |
| A*01 | 0 |  |  | 4 | 2,5 | 2.0 - 3.0 | 0 |  |  |
| A*01:01 | 0 |  |  | 8 | 2 | 2.0 - 2.25 | 0 |  |  |
| A*02 | 0 |  |  | 2 | 2 | 1.5 - 2.5 | 0 |  |  |
| A*02:01 | 0 |  |  | 18 | 2 | 1.0 - 2.0 | 0 |  |  |
| A*03 | 0 |  |  | 2 | 1 | 1.0 - 1.0 | 0 |  |  |
| A*03:01 | 0 |  |  | 4 | 2 | 1.75 - 2.0 | 0 |  |  |
| A*11:01 | 0 |  |  | 4 | 1,5 | 1.0 - 2.5 | 0 |  |  |
| A*23 | 0 |  |  | 2 | 3 | 2.5 - 3.5 | 0 |  |  |
| A*23:01 | 1 | 3 | 3.0 - 3.0 | 2 | 1,5 | 1.25 - 1.75 | 0 |  |  |
| A*24:02 | 0 |  |  | 8 | 1,5 | 1.0 - 3.0 | 1 | 2 | 2.0 - 2.0 |
| A*25 | 0 |  |  | 2 | 1,5 | 1.25 - 1.75 | 0 |  |  |
| A*25:01 | 0 |  |  | 2 | 1,5 | 1.25 - 1.75 | 1 | 2 | 2.0 - 2.0 |
| A*26 | 0 |  |  | 2 | 1 | 1.0 - 1.0 | 0 |  |  |
| A*26:01 | 0 |  |  | 5 | 2 | 1.0 - 2.0 | 0 |  |  |
| A*29 | 0 |  |  | 1 | 1 | 1.0 - 1.0 | 0 |  |  |
| A*31 | 0 |  |  | 2 | 2,5 | 1.75 - 3.25 | 0 |  |  |
| A*31:01 | 0 |  |  | 0 |  |  | 2 | 2,5 | 2.25 - 2.75 |
| A*32 | 0 |  |  | 1 | 2 | 2.0 - 2.0 | 0 |  |  |
| A*32:01 | 0 |  |  | 1 | 2 | 2.0 - 2.0 | 1 | 3 | 3.0 - 3.0 |
| A*66:01 | 0 |  |  | 1 | 1 | 1.0 - 1.0 | 0 |  |  |
| A*68:01 | 0 |  |  | 1 | 1 | 1.0 - 1.0 | 0 |  |  |
| A2 | 0 |  |  | 2 | 1,5 | 1.25 - 1.75 | 0 |  |  |
| A24 | 0 |  |  | 1 | 1 | 1.0 - 1.0 | 0 |  |  |
| B*07 | 0 |  |  | 1 | 1 | 1.0 - 1.0 | 0 |  |  |
| B*07:02 | 0 |  |  | 5 | 2 | 1.0 - 2.0 | 0 |  |  |
| B*08 | 0 |  |  | 6 | 1,5 | 1.0 - 2.75 | 0 |  |  |
| B*08:01 | 0 |  |  | 14 | 1 | 1.0 - 2.0 | 0 |  |  |
| B*13 | 0 |  |  | 1 | 1 | 1.0 - 1.0 | 0 |  |  |
| B*13:02 | 0 |  |  | 6 | 1 | 1.0 - 1.0 | 0 |  |  |
| B*14:02 | 0 |  |  | 1 | 1 | 1.0 - 1.0 | 0 |  |  |
| B*15 | 0 |  |  | 1 | 2 | 2.0 - 2.0 | 0 |  |  |
| B*15:01 | 0 |  |  | 1 | 1 | 1.0 - 1.0 | 0 |  |  |
| B*18:01 | 1 | 3 | 3.0 - 3.0 | 1 | 1 | 1.0 - 1.0 | 0 |  |  |
| B*27 | 0 |  |  | 2 | 2 | 2.0 - 2.0 | 0 |  |  |
| B*27:05 | 0 |  |  | 1 | 1 | 1.0 - 1.0 | 1 | 2 | 2.0 - 2.0 |
| B*35 | 0 |  |  | 1 | 2 | 2.0 - 2.0 | 0 |  |  |
| B*35:01 | 1 | 3 | 3.0 - 3.0 | 2 | 2 | 1.5 - 2.5 | 0 |  |  |
| B*37:01 | 0 |  |  | 2 | 2 | 1.5 - 2.5 | 0 |  |  |
| B*38:01 | 0 |  |  | 1 | 1 | 1.0 - 1.0 | 0 |  |  |
| B*39:01 | 0 |  |  | 1 | 2 | 2.0 - 2.0 | 0 |  |  |
| B*40:01 | 0 |  |  | 1 | 2 | 2.0 - 2.0 | 0 |  |  |
| B*41 | 0 |  |  | 1 | 1 | 1.0 - 1.0 | 0 |  |  |
| B*44 | 0 |  |  | 2 | 1 | 1.0 - 1.0 | 0 |  |  |
| B*44:02 | 0 |  |  | 6 | 1 | 1.0 - 1.0 | 0 |  |  |
| B*44:03 | 0 |  |  | 6 | 1 | 1.0 - 1.75 | 0 |  |  |
| B*49:01 | 0 |  |  | 2 | 2 | 2.0 - 2.0 | 0 |  |  |
| B*50:01 | 0 |  |  | 1 | 2 | 2.0 - 2.0 | 0 |  |  |
| B*51 | 0 |  |  | 1 | 1 | 1.0 - 1.0 | 0 |  |  |
| B*51:01 | 0 |  |  | 5 | 2 | 1.0 - 2.0 | 1 | 2 | 2.0 - 2.0 |
| B*52 | 0 |  |  | 1 | 2 | 2.0 - 2.0 | 0 |  |  |
| B*52:01 | 0 |  |  | 1 | 2 | 2.0 - 2.0 | 0 |  |  |
| B*55:01 | 0 |  |  | 2 | 2 | 1.5 - 2.5 | 1 | 2 | 2.0 - 2.0 |
| B*57:01 | 0 |  |  | 4 | 2 | 2.0 - 2.25 | 0 |  |  |
| B*58 | 0 |  |  | 1 | 4 | 4.0 - 4.0 | 0 |  |  |
| B*58:01 | 0 |  |  | 1 | 1 | 1.0 - 1.0 | 0 |  |  |
| B40 | 0 |  |  | 1 | 1 | 1.0 - 1.0 | 0 |  |  |
| C*01 | 0 |  |  | 0 |  |  | 1 | 2 | 2.0 - 2.0 |
| C*01:02 | 1 | 2 | 2.0 - 2.0 | 2 | 1,5 | 1.25 - 1.75 | 2 | 2 | 2.0 - 2.0 |
| C*02:02 | 0 |  |  | 5 | 1 | 1.0 - 1.0 | 1 | 1 | 1.0 - 1.0 |
| C*03 | 0 |  |  | 2 | 2,5 | 2.25 - 2.75 | 0 |  |  |
| C*03:03 | 1 | 3 | 3.0 - 3.0 | 2 | 1 | 1.0 - 1.0 | 0 |  |  |
| C*03:04 | 1 | 3 | 3.0 - 3.0 | 5 | 1 | 1.0 - 2.0 | 0 |  |  |
| C*04 | 0 |  |  | 2 | 2 | 1.5 - 2.5 | 0 |  |  |
| C*04:01 | 0 |  |  | 6 | 1 | 1.0 - 1.75 | 0 |  |  |
| C*05:01 | 0 |  |  | 2 | 1,5 | 1.25 - 1.75 | 1 | 2 | 2.0 - 2.0 |
| C*06 | 0 |  |  | 1 | 2 | 2.0 - 2.0 | 0 |  |  |
| C*06:02 | 0 |  |  | 2 | 2 | 1.5 - 2.5 | 0 |  |  |
| C*07:02 | 0 |  |  | 3 | 3 | 2.0 - 3.0 | 0 |  |  |
| C*12:03 | 1 | 2 | 2.0 - 2.0 | 0 |  |  | 0 |  |  |
| C*14:02 | 0 |  |  | 1 | 2 | 2.0 - 2.0 | 0 |  |  |
| C*15:02 | 0 |  |  | 1 | 1 | 1.0 - 1.0 | 0 |  |  |
| C*16 | 0 |  |  | 1 | 1 | 1.0 - 1.0 | 0 |  |  |
| C*17 | 0 |  |  | 0 |  |  | 1 | 1 | 1.0 - 1.0 |
| C*17:01 | 0 |  |  | 1 | 1 | 1.0 - 1.0 | 0 |  |  |
| DPB1*02:01 | 0 |  |  | 1 | 3 | 3.0 - 3.0 | 0 |  |  |
| DPB1*03:01 | 0 |  |  | 1 | 3 | 3.0 - 3.0 | 0 |  |  |
| DPB1*04:01 | 0 |  |  | 4 | 1 | 1.0 - 1.5 | 1 | 2 | 2.0 - 2.0 |
| DPB1*04:02 | 0 |  |  | 3 | 2 | 1.5 - 2.5 | 0 |  |  |
| DPB1*05:01 | 0 |  |  | 1 | 1 | 1.0 - 1.0 | 0 |  |  |
| DPB1*11:01 | 0 |  |  | 2 | 3 | 2.5 - 3.5 | 0 |  |  |
| DPB1*14:01 | 0 |  |  | 2 | 1,5 | 1.25 - 1.75 | 0 |  |  |
| DPB1*17 | 0 |  |  | 1 | 2 | 2.0 - 2.0 | 0 |  |  |
| DPB1*17:01 | 0 |  |  | 3 | 2 | 2.0 - 2.0 | 0 |  |  |
| DQ1 | 1 | 3 | 3.0 - 3.0 | 0 |  |  | 0 |  |  |
| DQA1*01:02 | 0 |  |  | 1 | 4 | 4.0 - 4.0 | 0 |  |  |
| DQA1*01:03 | 0 |  |  | 1 | 3 | 3.0 - 3.0 | 0 |  |  |
| DQA1*02:01 | 0 |  |  | 1 | 1 | 1.0 - 1.0 | 0 |  |  |
| DQA1*03.03 | 0 |  |  | 1 | 1 | 1.0 - 1.0 | 0 |  |  |
| DQA1*03:02 | 1 | 4 | 4.0 - 4.0 | 0 |  |  | 0 |  |  |
| DQA1*03:03 | 0 |  |  | 3 | 2 | 2.0 - 2.5 | 2 | 2,5 | 2.25 - 2.75 |
| DQA1*04:01 | 1 | 4 | 4.0 - 4.0 | 1 | 2 | 2.0 - 2.0 | 0 |  |  |
| DQA1*05:01 | 0 |  |  | 8 | 1,5 | 1.0 - 2.0 | 0 |  |  |
| DQA1*05:05 | 0 |  |  | 13 | 2 | 2.0 - 2.0 | 0 |  |  |
| DQB1*02 | 0 |  |  | 1 | 1 | 1.0 - 1.0 | 0 |  |  |
| DQB1*02:01 | 5 | 4 | 4.0 - 4.0 | 9 | 2 | 1.0 - 3.0 | 1 | 1 | 1.0 - 1.0 |
| DQB1*02:01:02 | 0 |  |  | 1 | 2 | 2.0 - 2.0 | 0 |  |  |
| DQB1*02:01~DQA1*05:01 | 2 | 3,5 | 3.25 - 3.75 | 5 | 1 | 1.0 - 2.0 | 0 |  |  |
| DQB1*02:02 | 2 | 4 | 4.0 - 4.0 | 5 | 2 | 1.0 - 3.0 | 0 |  |  |
| DQB1*03 | 0 |  |  | 2 | 1,5 | 1.25 - 1.75 | 0 |  |  |
| DQB1*03:01 | 8 | 3,5 | 3.0 - 4.0 | 15 | 2 | 1.0 - 2.5 | 0 |  |  |
| DQB1*03:01~DQA1*05:05 | 1 | 4 | 4.0 - 4.0 | 0 |  |  | 0 |  |  |
| DQB1*03:02 | 1 | 4 | 4.0 - 4.0 | 6 | 2,5 | 1.25 - 3.0 | 2 | 4 | 4.0 - 4.0 |
| DQB1*03:02~DQA1*03:01 | 0 |  |  | 2 | 2 | 2.0 - 2.0 | 0 |  |  |
| DQB1*03:03 | 2 | 3 | 2.5 - 3.5 | 3 | 3 | 2.5 - 3.5 | 0 |  |  |
| DQB1*04:02 | 0 |  |  | 2 | 1,5 | 1.25 - 1.75 | 1 | 2 | 2.0 - 2.0 |
| DQB1*04:02~DQA1*02:01 | 0 |  |  | 1 | 2 | 2.0 - 2.0 | 0 |  |  |
| DQB1*05 | 1 | 3 | 3.0 - 3.0 | 0 |  |  | 0 |  |  |
| DQB1*05:01 | 4 | 2,5 | 2.0 - 3.25 | 5 | 2 | 1.0 - 2.0 | 0 |  |  |
| DQB1*05:01~DQA1*01:01 | 0 |  |  | 1 | 4 | 4.0 - 4.0 | 0 |  |  |
| DQB1*05:02 | 0 |  |  | 2 | 3 | 2.5 - 3.5 | 0 |  |  |
| DQB1*05:03 | 0 |  |  | 1 | 1 | 1.0 - 1.0 | 0 |  |  |
| DQB1*06:02 | 1 | 2 | 2.0 - 2.0 | 6 | 1,5 | 1.0 - 2.75 | 0 |  |  |
| DQB1*06:02~ DQA1*01:02 | 0 |  |  | 1 | 1 | 1.0 - 1.0 | 0 |  |  |
| DQB1*06:03 | 1 | 4 | 4.0 - 4.0 | 10 | 2 | 1.25 - 3.0 | 0 |  |  |
| DQB1*06:03~DQA1*01:03 | 0 |  |  | 1 | 2 | 2.0 - 2.0 | 0 |  |  |
| DQB1*06:04 | 0 |  |  | 4 | 1 | 1.0 - 1.25 | 0 |  |  |
| DRB1*01:01 | 0 |  |  | 1 | 2 | 2.0 - 2.0 | 0 |  |  |
| DRB1*01:02 | 0 |  |  | 2 | 3 | 2.5 - 3.5 | 0 |  |  |
| DRB1*03:01 | 0 |  |  | 2 | 1,5 | 1.25 - 1.75 | 0 |  |  |
| DRB1*04:01 | 1 | 3 | 3.0 - 3.0 | 4 | 2 | 2.0 - 2.25 | 0 |  |  |
| DRB1*04:04 | 0 |  |  | 2 | 1,5 | 1.25 - 1.75 | 0 |  |  |
| DRB1*07:01 | 0 |  |  | 9 | 2 | 1.0 - 3.0 | 2 | 1,5 | 1.25 - 1.75 |
| DRB1*08:01 | 0 |  |  | 2 | 1,5 | 1.25 - 1.75 | 0 |  |  |
| DRB1*09:01 | 0 |  |  | 1 | 2 | 2.0 - 2.0 | 0 |  |  |
| DRB1*10:01 | 0 |  |  | 1 | 2 | 2.0 - 2.0 | 0 |  |  |
| DRB1*12:01 | 0 |  |  | 2 | 3 | 2.5 - 3.5 | 0 |  |  |
| DRB1*13:03 | 1 | 2 | 2.0 - 2.0 | 0 |  |  | 0 |  |  |
| DRB1*14:01 | 0 |  |  | 1 | 3 | 3.0 - 3.0 | 0 |  |  |
| DRB1*15:01 | 0 |  |  | 3 | 1 | 1.0 - 1.5 | 0 |  |  |
| DRB3*01:01 | 0 |  |  | 3 | 2 | 1.5 - 2.0 | 0 |  |  |
| DRB3*02 | 0 |  |  | 1 | 3 | 3.0 - 3.0 | 0 |  |  |
| DRB3*02:02 | 0 |  |  | 3 | 1 | 1.0 - 2.5 | 1 | 2 | 2.0 - 2.0 |
| DRB4 | 0 |  |  | 2 | 1,5 | 1.25 - 1.75 | 0 |  |  |
| DRB4*01:01 | 0 |  |  | 3 | 2 | 1.5 - 2.0 | 0 |  |  |
| DRB4*01:03 | 5 | 3 | 3.0 - 4.0 | 17 | 2 | 1.0 - 3.0 | 0 |  |  |
| DRB5 | 0 |  |  | 1 | 3 | 3.0 - 3.0 | 0 |  |  |
| DRB5*01 | 0 |  |  | 1 | 3 | 3.0 - 3.0 | 0 |  |  |
| DRB5*01:01 | 0 |  |  | 9 | 2 | 1.0 - 2.0 | 1 | 1 | 1.0 - 1.0 |
| DRB5*02:02 | 0 |  |  | 1 | 2 | 2.0 - 2.0 | 0 |  |  |
