# Supplementary material for: Impact of Transient and Persistent Donor-Specific Antibodies in Lung Transplantation
Source: Transpl Int. 2024 May 8;37:12774. doi: 10.3389/ti.2024.12774 (PMC11110840; doi:10.3389/ti.2024.12774)
Supplement: Supplementary file 2 [file Table2.docx]

| Patients’ characteristics | | | | | |
| --- | --- | --- | --- | --- | --- |
|  | | transient n=154 | peristent n=38 | recurrent n=13 | p-value |
| Female (n, %) | | 63 (41%) | 6 (16%) | 4 (31%) | 0.012 |
| Age in years (median, IQR) | | 57 (43, 62) | 53 (35, 60) | 55 (48, 60) | 0.3 |
| Type of TX (n, %) | DLuTx | 152 (99%) | 37 (97%) | 13 (100%) | 0.6 |
|  | SLuTx right | 1 (0.6%) | 1 (2.6%) | 0 (0%) |  |
|  | SLuTx left | 1 (0.6%) | 0 (0%) | 0 (0%) |  |
| Underlying diagnosis (n, %) | Obstructive | 65 (42%) | 12 (32%) | 7 (53%) | 0.11 |
|  | Restrictive | 48 (31%) | 13 (34%) | 4 (31%) |  |
|  | Vascular | 8 (5.2%) | 0 (0%) | 1 (7.7%) |  |
|  | Septic | 26 (17%) | 11 (29%) | 1 (7.7%) |  |
|  | Others | 7 (4.5%) | 2 (5.3%) | 0 (0%) |  |
| UAGs (n, %) | | 10 (6.9%) | 1 (3.1%) | 0 (0%) | 0.9 |
| crossmatch positive (n, %) | | 1 (0.7%) | 0 (0%) | 0 (0%) | >0.9 |
| High grade ACR (n, %) | | 3 (1.9%) | 4 (11%) | 1 (7.7%) | 0.031 |
| High grade LB (n, %) | | 4 (2.6%) | 4 (11%) | 1 (7.7%) | 0.053 |
| Immuno-  supression (n, %) | Ciclosporin | 4 (2.6%) | 0 (0%) | 0 (0%) | >0.9 |
|  | Tacrolimus | 149 (97%) | 37 (100%) | 12 (100%) |  |
| CMV risk (n, %) | D+/R- | 38 (25%) | 16 (42%) | 4 (31%) | 0.3 |
|  | D+/R+ | 57 (37%) | 7 (18%) | 4 (31%) |  |
|  | D-/R+ | 42 (27%) | 13 (34%) | 3 (23%) |  |
|  | D-/R- | 16 (10%) | 2 (5.3%) | 2 (15%) |  |
| AMR (n, %) | | 11 (7.1%) | 9 (24%) | 3 (23%) | 0.005 |
| HLA class I (n, %) | | 95 (62%) | 27 (71%) | 11 (85%) | 0.2 |
| HLA class II (n, %) | | 99 (64%) | 35 (92%) | 9 (69%) | 0.002 |
| DSA against HLA - A (n, %) | | 48 (31%) | 18 (47%) | 3 (23%) | 0.13 |
| DSA against HLA - B (n, %) | | 50 (32%) | 20 (53%) | 5 (38%) | 0.071 |
| DSA against HLA - C (n, %) | | 26 (17%) | 10 (26%) | 7 (54%) | 0.007 |
| DSA against HLA - DQ (n, %) | | 78 (51%) | 32 (84%) | 6 (46%) | <0.001 |
| DSA against HLA - DP (n, %) | | 10 (6.5%) | 5 (13%) | 3 (23%) | 0.053 |
| DSA against HLA - DR (n, %) | | 34 (22%) | 16 (42%) | 6 (46%) | 0.012 |
| mean MFI intensity score (median, IQR) | | 2.00 (1.00-2.00) | 2.53 (2.00-2.96) | 1.50 (1.20-2.00) | <0.001 |
| CLAD (n, %) | | 30 (19%) | 17 (45%) | 5 (38%) | 0.003 |

Supplementary table 2: Patients characteristics for transient, persistent, and recurrent dnDSA

Abbreviations: TX = transplantation, DLuTx = double lung transplantation, SLuTx = single lung transplantation, HLA = human leukocyte antigen, UAG = unacceptable antigen, ACR = acute cellular rejection, LB = lymphocytic bronchiolitis, D = donor, R = recipient, AMR = antibody mediated rejection, DSA = donor specific antibody, MFI = mean fluorescence intensity, CLAD = chronic lung allograft dysfunction
