# Supplementary material for: Impact of Transient and Persistent Donor-Specific Antibodies in Lung Transplantation
Source: Transpl Int. 2024 May 8;37:12774. doi: 10.3389/ti.2024.12774 (PMC11110840; doi:10.3389/ti.2024.12774)
Supplement: Supplementary file 3 [file Table3.docx]

Supplementary Table 3: Treated patients

| Patients | dnDSA | MFI class | AMR | ECP | IAS | Plasmapheresis | Daratumumab | IVIG | ATG | CLAD | Death |
| --- | --- | --- | --- | --- | --- | --- | --- | --- | --- | --- | --- |
| patnr001 | transient | 2 | yes | no | no | yes | no | yes | no | no | yes |
| patnr002 | persistent | 3 | yes | yes | no | no | no | no | no | yes | yes |
| patnr003 | transient | 1 | yes | no | no | no | no | no | no | no | yes |
| patnr004 | persistent | 4 | yes | yes | yes | no | yes | no | no | yes | yes |
| patnr005 | transient | 4 | yes | yes | yes | no | no | no | yes | no | yes |
| patnr006 | persistent | 4 | yes | yes | yes | no | no | no | no | yes | yes |
| patnr007 | persistent | 4 | yes | no | yes | no | yes | no | no | yes | yes |
| patnr008 | transient | 3 | yes | no | yes | no | yes | no | no | yes | no |
| patnr009 | transient | 3 | yes | no | yes | no | no | no | yes | no | no |
| patnr010 | transient | 3 | yes | no | yes | yes | no | no | no | no | yes |
| patnr011 | transient | 4 | yes | yes | yes | no | no | no | no | no | no |
| patnr012 | transient | 4 | yes | yes | yes | no | no | no | no | no | yes |
| patnr013 | transient | 4 | yes | yes | yes | no | no | no | no | yes | yes |
| patnr014 | transient | 2 | yes | yes | no | no | no | no | no | no | no |
| patnr015 | transient | 4 | yes | no | yes | yes | yes | no | yes | no | no |
| patnr016 | persistent | 4 | yes | no | yes | no | no | yes | no | yes | yes |
| patnr017 | transient | 4 | yes | yes | no | no | no | no | no | yes | no |
| patnr018 | persistent | 2 | yes | yes | no | no | no | no | no | yes | yes |
| patnr019 | persistent | 4 | yes | yes | no | no | no | no | no | yes | yes |
| patnr020 | transient | 4 | yes | no | yes | no | no | no | no | yes | yes |
| patnr021 | transient | 4 | yes | no | yes | no | no | no | no | no | no |
| patnr022 | persistent | 3 | yes | no | yes | no | no | no | no | no | yes |

Abbreviations: dnDSA = denovo donor specific antibodies, MFI = mean fluorescence intensity, AMR = antibody mediated rejection, ECP = extracorporeal photopheresis, IAS = immunoadsorption, IVIG = intravenous immunoglobulin, ATG = anti-thymocyte globulin, CLAD = chronic lung allograft dysfunction
